# Unusual and atypical cyclooxygenase reactions

**DOI:** 10.1016/j.jbc.2026.111247

**Published:** 2026-02-05

**Authors:** Claus Schneider, Alan R. Brash

**Affiliations:** Department of Pharmacology and the Vanderbilt Institute of Chemical Biology, Vanderbilt University School of Medicine, Nashville, Tennessee, USA

**Keywords:** arachidonic acid, aspirin, cyclooxygenase, drug action, eicosanoid, enzyme catalysis, enzyme functional plasticity, fish oil, oxylipin, prostaglandin, stereocontrol

## Abstract

Mechanistic studies have yielded novel prostaglandin analogs and acyclic products initially of interest in understanding cyclooxygenase (COX) structure and function, later found *in vivo* and of interest because of unique biological activities. Beyond arachidonic acid, fatty acid substrates span from 18 to 22 carbons and may contain ester/amide modification or epoxide/hydroxy moieties at the first double bond. Stereocontrol with the unconventional substrates remains largely intact although cyclization may be diverted or halted altogether, and catalysis proceeds with the insertion of one, two, or three molecules of oxygen into substrates. A switch in stereochemistry at the 15-carbon occurs in a natural COX from coral and has received attention upon aspirin treatment of COX-2. The latter produces 15*R*-hydroxyeicosatetraenoic acid and analogs from other fatty acids that may be further oxygenated by lipoxygenases. Functional plasticity in COX catalysis has enabled the formation and discovery of a host of novel eicosanoids and provided mechanistic insight into the COX reaction mechanisms.

It can help with the understanding of enzyme mechanism to explore the range of catalysis beyond the transformations of the regular physiological substrates. This can stretch the system to the edge of what is possible and provide limits to the proposed mechanisms. In the case of prostaglandin H (PGH) synthase (cyclooxygenase [COX]), this has filled in details of substrate binding and activation energy in hydrogen abstraction, and among other innovations, extended the conventional range of polyunsaturated fatty acid (PUFA) oxygenations. Herein, we will briefly outline conventional PG synthesis, then consider other fatty acid substrates, the byproducts of COX catalysis, and the transformations of hydroxyeicosatetraenoic acids (HETEs) and epoxy-fatty acids in a physiological context.

## Conventional PUFAs for PG synthesis

**Figure 1:**
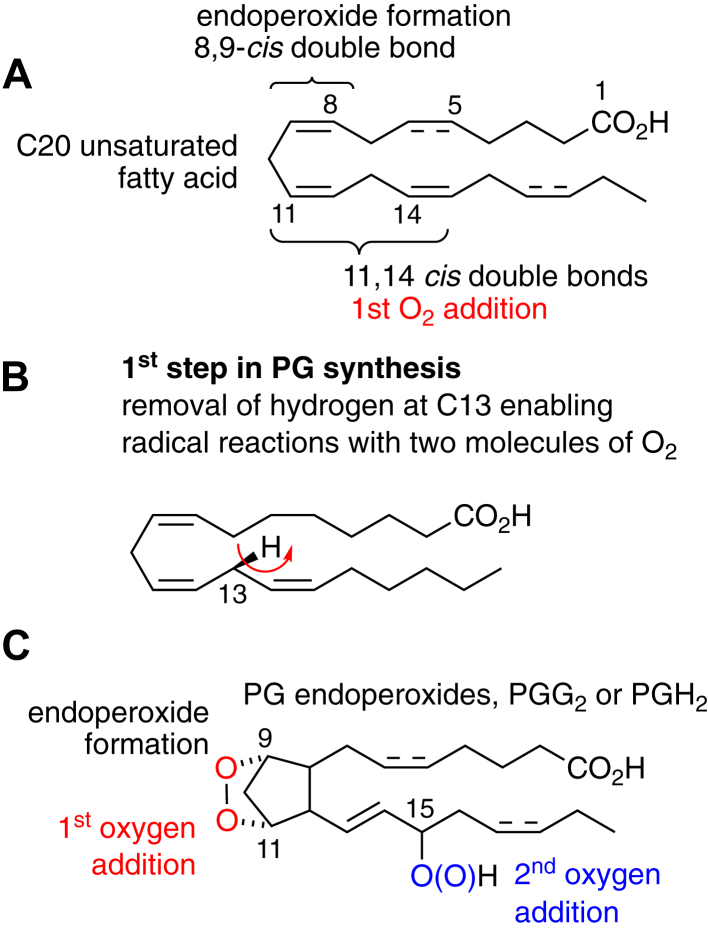
**Basic elements of prostaglandin (PG) endoperoxide synthesis**. *A*, conventional PG synthesis uses a C20 unsaturated fatty acid as a substrate. As illustrated, three double bonds are required to complete PG endoperoxide formation; two more are optional and indicated with hashed bonds. The most common substrate, arachidonic acid (20:4ω6), has the three essential bonds and the fourth on carbons 5,6 in the top fatty acid chain. *B*, the first step in PG biosynthesis is the removal of a hydrogen atom from the 13-carbon. This creates a fatty acid–free radical that reacts consecutively with two molecules of O_2_. *C*, the end products of cyclooxygenase catalysis are PGG_2_ or PGH_2_ (15-hydroxy); their chemical structures contain the first and second O_2_ molecules used for catalysis.

This is well covered in previous reviews and in other contributions to this commemorative series (1). The essential elements for COX catalysis forming PGs are three *cis*-double bonds in the 8,9-, 11,12-, and 14,15-positions of a 20-carbon fatty acid (Fig. 1). The *cis*-double bonds in the 5,6-position, as in the prototypical substrate, arachidonic acid (AA; 20:4ω6), and the additional 17,18-*cis* double bond in eicosapentaenoic acid (EPA; 20:5ω3) are not required and not affected in the reaction. Catalysis is initiated by hydrogen removal from C-13, the methylene flanked by the 11,12- and 14,15-*cis* double bonds, and provides a carbon-centered radical that enables the reaction with the O_2_ substrate, PG ring formation, and a second reaction with O_2_ to yield PGG_2_, the PG 9,11-endoperoxide, 15-hydroperoxide, that undergoes reduction to the 15-hydroxide (PGH_2_) to complete the catalytic cycle. PGH_2_ is the physiological substrate for enzymatic metabolism by various synthases to effector PGs like PGE_2_, thromboxane, and prostacyclin.

It is worth noting that the first PGs to be structurally characterized were PGE_1_ and PGF_1α_ (2). This came about because sheep seminal vesicles represented the biological source for the lipids analyzed, and the tissue is rich in dihomo-γ-linolenic acid (20:3ω6). This atypical abundance of 20:3ω6 substrate and the corresponding 1-series PGs is matched to some extent in humans, as evidenced by the similar concentrations in seminal plasma of 1- and 2-series PGEs, particularly 19-hydroxy-PGE_1_ and 19-hydroxy-PGE_2_ (3, 4, 5), and found in concentrations in the order of 100 μg/ml. As an aside, it is fascinating to recognize that the 19-hydroxy-PGs arise *via* the actions of a cytochrome P450 (CYP4F8) that specializes in 19-hydroxylation of the unstable PGH_1_ and PGH_2_ endoperoxides (6).

### C18 fatty acid substrates

**Figure 2:**
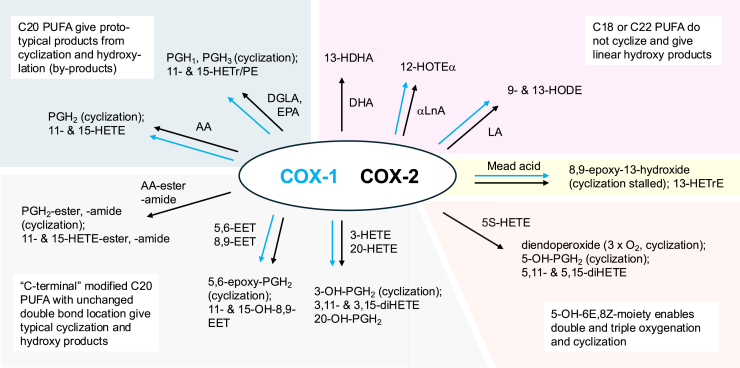
**Reactions of COX-1 and COX-2 with polyunsaturated fatty acids and their modified analogs**. Reactions are grouped into “normal” catalysis with AA, EPA, and DGLA substrates yielding PGH_2_ and the 1- and 3-series analogs, C-18 and C-22 substrates that do not cyclize and give hydroxylated products, Mead acid where cyclization is stalled; and carboxylate and methyl terminal-modified PUFA, including epoxides and hydroxides, that largely undergo cyclization to the respective PGH_2_ analogs or, in the case of 5-HETE, in addition undergo triple oxygenation to yield a diendoperoxide. Additional, but not all, minor products are noted. *Arrows* in *blue* and *black* indicate reactions catalyzed by COX-1 or COX-2, respectively. AA, arachidonic acid; COX, cyclooxygenase; DGLA, dihomo-γ-linolenic acid; EPA, eicosapentaenoic acid; HETE, hydroxyeicosatetraenoic acid; PGH, prostaglandin H.

Early studies by Hamberg and Samuelsson (7) on the oxygenation of unsaturated fatty acids by sheep vesicular glands included the metabolism of linoleic acid (18:2ω6), which is oxygenated predominantly to 9-hydroxy-octadecadieonic acid (82%) and the remainder to the 13-hydroxy isomer (Fig. 2). These were later characterized by chiral analysis as mainly 9*R* and 13*S*, respectively (8). The 9*R*-oxygenation of linoleate matches the 11*R*-oxygenation of arachidonate that initiates PG synthesis (9, 10, 11). Later work by Smith *et al*. using recombinant human COX-1 and COX-2 showed that linoleate is a relatively weak substrate for COX-1, whereas with COX-2, its oxygenation efficiency achieves about 60% of the rate with dihomo-γ-linolenic acid and AA (12).

Given the understanding of the COX metabolism of linoleic acid and AA, the results with α-linolenic acid (18:3ω3) produced an unexpected product as described by Smith *et al*.; the anticipated product would be the 9*R*-hydroxy isomer, yet catalysis produced 12-hydroxy-octadecatrienoic acid (Fig. 2) (12). This was rationalized as a kink in the bound substrate redirecting the initiating hydrogen abstraction from *bis*-allylic C11 to the *bis*-allylic 14-carbon, essentially a frameshift in oxygenation specificity. This concept was confirmed and illustrated in detail with the advent of COX-2 crystal structures and the analysis of fatty acid binding (13), and given a prominence in another contribution to this commemorative series (Malkowski, this series).

### C20:3ω9 and C20:5ω3 fatty acid substrates

**Figure 3:**
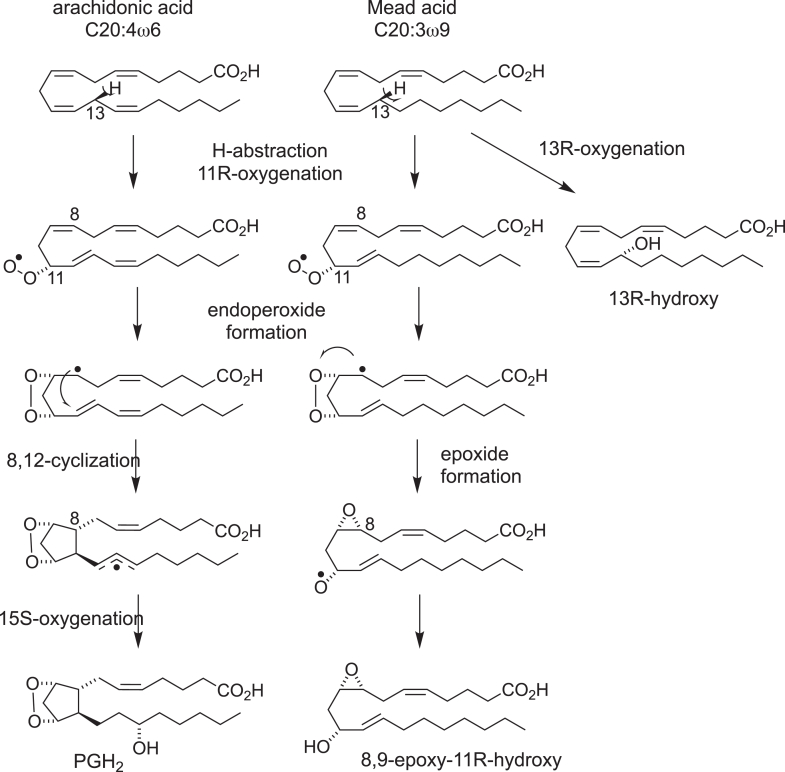
**Comparison of the COX reaction with AA and** M**ead acid**. The initial steps of H-abstraction and 11*R*-oxygenation are identical with both substrates, but the C-8 radical proceeds differently: with AA, the prostane ring is formed by reaction of C-8 with C-12 in a 5-exo-cyclization, followed by a second oxygenation at C-15 to yield PGH_2_. With Mead acid, cyclization does not occur, and the C-8 radical reacts with the peroxide to form an 8,9-epoxide and 11-hydroxide. An additional product is the 13*R*-hydroxide resulting from antarafacial oxygenation following C13 H-abstraction. AA, arachidonic acid; COX, cyclooxygenase; PGH, prostaglandin H.

Mead acid (20:3ω9) accumulates in essential fatty acid deficiency as the lack of linoleate (18:2ω6) substrate is replaced by oleate (18:1ω9), and the fatty acid desaturase and elongase machinery creates a PUFA with no ω3 or ω6 double bond (14). This presents an interesting potential substrate for COX catalysis, primarily because it lacks the usual *bis*-allylic methylene at C13. The outcome was explored in at least a couple of mechanistic studies, which found relatively slow oxygenation of the substrate (15, 16). Elliott *et al*. identified 13-hydroxy-20:3 as the main product along with several minor derivatives, including the 11-hydroxy isomer, and notably, no cyclized products (Fig. 2). Based on products recovered, it appears that the initial C13 hydrogen abstraction proceeds as in the reaction with AA but with minor follow-up with the usual 11*R*-oxygenation (Fig. 3). Instead, the main outcome is direct oxygenation on the 13-carbon. Oliw *et al*. reanalyzed the products from Mead acid and provided significant new insights (17). The 13-hydroxy product was shown to be 13*R* in chirality, antarafacial to the expected 13 pro-*S* hydrogen abstraction. More pointedly, evidence for partial completion of the 9,11-endoperoxide was deduced from identification of an 8,9-epoxy-11-hydroxy-eicosadienoic acid (Fig. 3).

Mead acid transformation to the 8,9-epoxy-11-hydroxy product is an interesting case of stalled COX catalysis, where the 9,11-peroxide-8-carbinyl radical is unable to react with the 12,13-double bond to close the prostane ring. Instead, the C-8 radical attacks the peroxide to yield an 8,9-epoxide and 11-hydroxide (Fig. 3). This product bears a close mechanistic resemblance to a later reaction in which mutant COX-2 enzymes formed similar diepoxyhydroxy products as a result of stalled catalysis (18). Alternatively, but not proven experimentally, the Mead acid–derived C8 carbon radical might have reacted with O_2_ to form a C8 peroxyl radical prior to reaction with C12, a reaction that has been shown to be preferred in the COX-2 transformation of 5-HETE (19), see later. The initial Mead acid incubations, however, were conducted with a COX-1 preparation (seminal vesicles), and it is conceivable that an 8,12-cyclization, either directly or through a peroxide, would only be accomplished in the larger COX-2 active site. An analysis of the reaction of COX-2 with radiolabeled Mead acid gave 13- and 11-hydroxyeicosatrienoic acids as major products and also showed more polar eluting material that might have included cyclized products (17). Notably, the “stalled” 8,9-epoxy-11-hydroxyeicosadienoic acid was not among the identified products, suggesting that its C8 radical precursor may have yielded cyclized products that were left uncharacterized (17).

Minor stalled or incomplete catalysis does occur even with AA and EPA. Although PGH_*2*_ and PGH_3_ are major products, detection of monohydroxy derivatives and 13-hydroxy-PGH endoperoxides is reported (9, 20, 21). Formation of the 13-hydroxy-PGH products entails oxygenation of the C13–C15 allyl radical at C13 rather than the normal oxygenation on the 15-carbon (Fig. 3), and it is remarkable that this does not occur more prominently.

### C22 substrates

An early article, currently cited around 500 times, reported no significant PG-related synthesis from docosahexaenoic acid (DHA) by COX (COX-1, sheep seminal vesicles), whereas DHA inhibited AA metabolism with a *K*_*i*_ of 0.36 μM (22). Transformation of DHA in other tissue preparations also found minimal COX-related metabolism (20, 23). Later work by Smith *et al*. using recombinant human COX-1 and COX-2 did establish low rates of oxygen uptake in metabolism of DHA (10–20% of the rate with arachidonate) with formation of a 13-hydroxy-DHA product (Fig. 2) (12, 24, 25). Of note, inhibition of AA metabolism by DHA with human COX enzymes was far less marked than in the earlier study, and particularly, COX-2 was minimally affected (25): DHA (100 μM) caused only ∼50% inhibition of O_2_ uptake associated with 20 μM AA metabolism, and in a later study, 5 μM DHA had no appreciable effect (24).

Aspirin-treated COX-2 exhibited further reduced activity with DHA as a substrate, forming a 1:3 mixture of 13-hydroxydocosahexaenoic acid (13-HDHA) and 17-HDHA at 5% the rate of AA transformation (24). Hydroxy-DHA derivatives (from COX or lipoxygenase [LOX] metabolism) can be substrates for formation of keto derivatives, detected from 22:5ω3 (EPA) and 22:6ω3 (DHA) as “EFOX,” electrophilic oxo oxylipins associated with inflammation (26).

## Modified PUFA as substrates: epoxides and hydroxides

**Figure 4:**
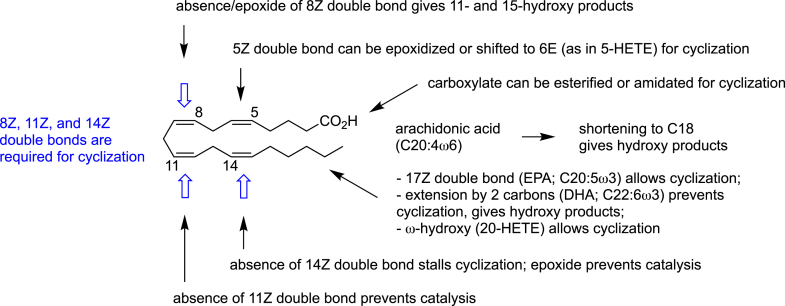
**Role of double bonds and carboxyl and methyl terminal modifications of AA in normal *versus* atypical catalysis in COX-2**. “Cyclization” refers to the formation of a prostaglandin endoperoxide or equivalent product, and only major effects are illustrated. Many of the modifications yield poor substrates. AA, arachidonic acid; COX, cyclooxygenase.

Besides linear PUFA with various chain lengths and location and number of methylene interrupted *cis* double bonds, other, more prominent modifications of PUFA structure are also tolerated for substrates, especially in COX-2 catalysis, which features a larger COX active site than COX-1 (Fig. 4) (27).

The first COX substrate to be identified that was not a simple PUFA was 5,6-epoxyeicosatrienoic acid 5,6-EET), the 5,6-epoxide of AA (28). 5,6-EET was an “obvious” modified substrate to test since the 8,9-, 11,12-, and 14,15-double bonds are still available, and these are the double bonds participating in COX catalysis (Figs. 3 and 4). Oxygenation of 5,6-EET with ram seminal vesicles, a source of abundant COX-1 activity, yielded two stereoisomers of 5-hydroxy-PGI_1_, namely (5*R*,6*R*)- and (5*S*,6*S*)-5-hydroxy-PGI_1_ that were suggested to be derived from a 5,6-epoxy-PGG/H_2_ or a 5,6-epoxy-PGF_1α_ intermediate (28, 29). A similar transformation of the 5,6-EET hydrolysis product, 5,6-dihydroxyeicosatrienoic acid (5,6-DiHET), yielded 5,6-dihydroxy-PGF_1α_ and 5,6-dihydroxy-PGE_1_ (30).

8,9-EET was shown to undergo stereospecific metabolism by COX-1, with the 8*S*,9*R*-epoxide yielding the corresponding 11*R*-hydroxy metabolite as the sole product, whereas the 8*R*,9*S*-epoxide formed both 11- and 15-hydroxy metabolites (31). 11,12-EET was a very poor substrate for both COX enzymes, and 14,15-EET did not react with either (Fig. 4) (32). 8,9-EET and 14,15-EET are also inhibitors of COX-1 *in vitro*, comparable in potency to ibuprofen, but whether this extends to inhibition *in vivo* is unclear (33).

3*R*-HETE was identified as a product of incomplete β-oxidation of AA by the fungus *Dipodascopsis uninucleata* (34). As a substrate for both COX-1 and COX-2, it was converted with about 10-fold lesser catalytic efficiency than AA, yielding the expected 3-OH-PGH_2_ product (Fig. 3) (35).

The renal vasoconstrictor 20-HETE is converted by COX to 20-hydroxy-PGH_2_ (Fig. 3) (36). Although 20-HETE has the same arrangement of double bonds as its parent AA, the formation of a PGH_2_ analog is somewhat unexpected, considering the spatial constraints in the upper channel of the COX binding site where the ω-end of the fatty acid is bound. The bottom of the channel is formed by Gly-533, and mutation to alanine nearly abolished the reaction with the C20 substrate AA, whereas the reaction with the C18 substrates α-linolenic acid and stearidonic acid was almost unchanged (37). This indicated that there is little free space near the terminal methyl group of AA, and it is unclear how its extension by a hydroxyl enables binding of 20-HETE in a configuration that allows oxygenation and double cyclization to the respective PGH_2_ product (36). The product analyses by TLC showed metabolites of varying polarities, with the most polar designated as 20-hydroxy-PG-endoperoxides (and 20-OH-PGF_2α_ after reduction); the nature of the additional products remains unresolved (36). Strikingly, the 20-hydroxy-endoperoxides (shown to be unstable after extraction during the first 30 min at room temperature) exhibited the most potent vasoconstrictor activity on rat aortic rings. Authentic stable 20-hydroxy-PGs did not elicit contraction (36). Altogether, this is a fascinating study with a biosynthesis by P450, followed by COX, which is opposite in order to the formation of 20-hydroxy-PGs in seminal plasma (6).

It is likely that, at some point in the early days of using seminal vesicles as a source of COX activity and prior to the discovery of COX-2, 5-HETE (with the 8-, 11-, and 14*-cis* double bonds available as in AA) was also tested as a potential COX substrate—it is an obvious candidate. As it turned out, 5-HETE, in contrast to 5,6-EET and 5,6-DiHET, is not a substrate for COX-1, that is, the enzyme present in seminal vesicles. 5-HETE, however, is a selective substrate for COX-2, and the enzyme is also stereoselective for the transformation of the 5*S*-enantiomer, the product of the 5-LOX reaction with AA, whereas the 5*R*-enantiomer is a very poor substrate (19). The difference in COX isoform selectivity between 5,6-EET and 5,6-DiHET *versus* 5-HETE is likely because of the rigidity of the conjugated diene in 5-HETE, preventing access to the COX-1 active site.

**Figure 5:**
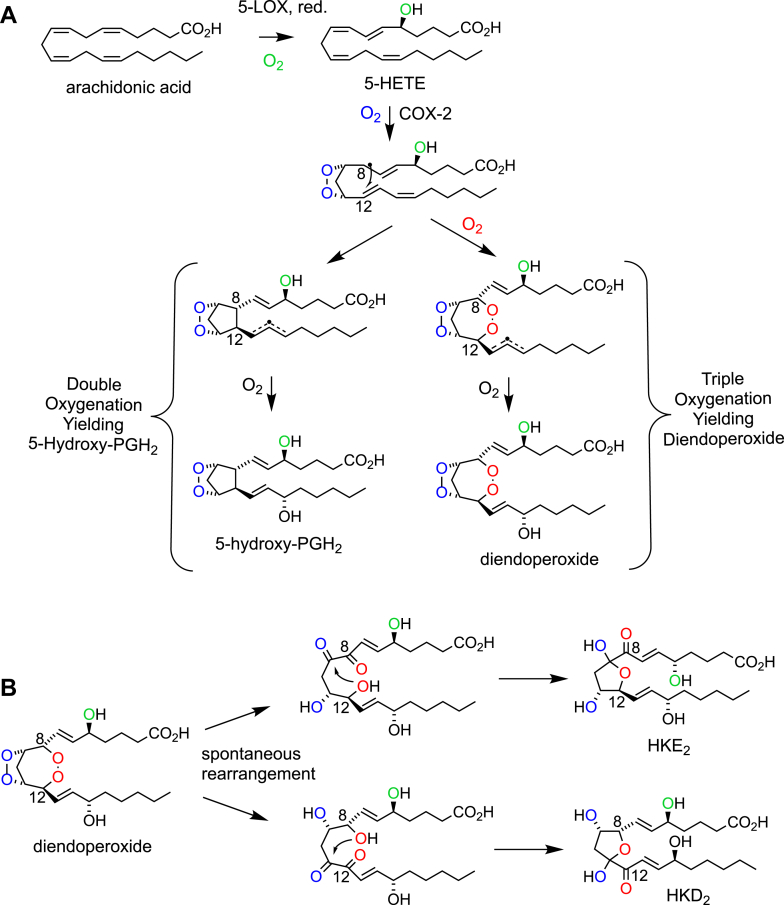
**The COX-2 reaction with 5-HETE**. A: The 5-LOX product 5-HETE yields either 5-hydroxy-PGH_2_ or a diendoperoxide, depending on whether two or three molecules of oxygen are inserted during catalysis. A key intermediate in determining catalytic outcome is a 9,11-peroxy-8-carbinyl radical that either reacts directly with C-12 to close the prostane ring or reacts with oxygen to a peroxyl before reacting with C-12. *B*, the peroxides of the 5-HETE/COX-2-derived diendoperoxide spontaneously rearrange into 1,2-diketo/-dihydroxy moieties that cyclize to yield the two hemiketal eicosanoids, HKE_2_ and HKD_2_. COX, cyclooxygenase; HETE, hydroxyeicosatetraenoic acid; HKD, hemiketal D; HKE, hemiketal E; LOX, lipoxygenase; PGH, prostaglandin H.

The major product of the COX-2 reaction with 5-HETE is a diendoperoxide in which the 8,12-bond of the prostane ring is expanded by a peroxide linking the two carbons, resulting in formation of an unusual 7-membered ring (19). The diendoperoxide carries a 15-hydroxyl like PGH_2_ in addition to the 5-hydroxyl from the 5-HETE substrate (Fig. 5*A*). Thus, COX-2 reacts 5-HETE with three molecules of oxygen rather than with two molecules when forming PGH_2_ from AA or any of its derivatives. Notably, the third oxygenation occurring at C-15 proceeds with the same 15*S* stereospecificity as in the formation of PGH_2_ (38). A second, albeit less abundant, product in the COX-2 reaction with 5-HETE is 5-hydroxy-PGH_2_, the intuitively expected product when using “5-hydroxy-AA” (*i*.*e*., 5-HETE) instead of AA as a substrate (Fig. 5*A*) (39). The 5-OH-PGH_2_ endoperoxide rearranges spontaneously to 5-OH-PGD_2_ and 5-OH-PGE_2_ and undergoes chain cleavage to 5,12-di-hydroxyheptadecatrienoic acid, equivalent to 12-hydroxyheptadecatrienoic acid formation from PGH_2_ (39). Other catalytic (by-)products are the 5-hydroxy analogs of 11- and 15-HETE, that is, 5,11- and 5,15-DiHETE (40). Thus, in the course of the reaction with 5-HETE, a key intermediate, the 9,11-endoperoxide-8-carbinyl radical, undergoes either direct cyclization with C-12 to form 5-OH-PGH_2_ or an unusual oxygen addition prior to reaction with C-12, ultimately yielding the diendoperoxide (Fig. 5*A*). This is another instance, in addition to Mead acid transformation, where the C8 carbon radical is a crucial mechanistic intermediate and where its further reaction decides between catalytic outcomes. Whether the enzyme exerts active control over this reaction, that is, the insertion of two *versus* three molecules of oxygen during the reaction with 5-HETE, and whether the outcome can be controlled by extrinsic factors, thereby shifting the balance of double *versus* triple oxygenated products, is not known.

The diendoperoxide is chemically more stable than PGH_2_ or 5-OH-PGH_2_ but still undergoes rearrangement of the peroxide moieties into keto-/hydroxy functional groups that yield the final two hemiketal (HK) eicosanoids, HKD_2_ and HKE_2_ (Fig. 5*B*) (41, 42). HKD_2_ and HKE_2_ are potent proangiogenic lipids *in vitro* and *in vivo* and also inhibit platelet aggregation (43, 44). A biological role for 5-OH-PGs has not yet been uncovered (39).

## Carboxylate-modified PUFA as COX-2 selective substrates

The COX-2 active site is larger than that of COX-1 (45) and anchoring of the substrate at the entrance of the channel is less dependent on the carboxylate ion pairing with an arginine residue at the channel entrance (46, 47). These factors contribute to COX-2 accepting amide and ester derivatives of PUFA as substrates. For example, anandamide (the ethanolamide of AA) and 2-arachidonyl-glycerol are efficient substrates for COX-2 oxygenation, yielding the corresponding amide or ester derivatives of PGH_2_, respectively (48, 49). Docosahexaenoylethanolamide (DHEA) is converted by human COX-2 into the previously unknown metabolites, 13- and 16-hydroxy-DHEA (13- and 16-HDHEA, respectively). These products were also produced by lipopolysaccharide-stimulated RAW264.7 macrophages incubated with DHEA. COX metabolism of fatty acid amides and esters is reviewed in this series (50).

Crystal structures showing binding of modified PUFA in the COX-2 active site do not show occupation of the so-called side pocket by the glycerol or ethanolamide moieties, respectively (51). The side pocket in the COX-2 fatty acid binding channel is accessible by the replacement of Ile523 in COX-1 with a valine in COX-2 and forms the structural basis for COX-2 selective inhibition (52, 53), but its biochemical role in COX catalysis or substrate binding, if any, has yet to be revealed.

## The effect of aspirin on COX catalysis

**Figure 6:**
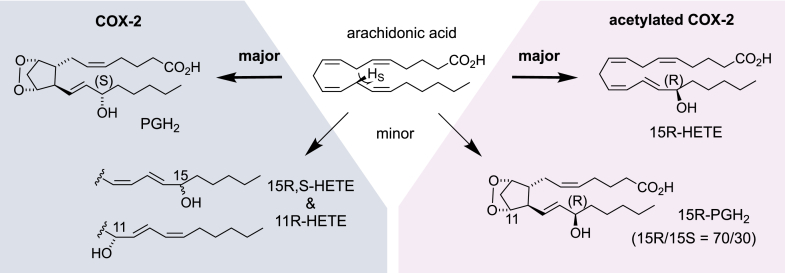
**The effect of aspirin on COX-2 catalytic activity**. Aspirin-mediated acetylation of Ser-530 of COX-2 shifts the major catalytic outcome away from PGH_2_ to 15*R*-HETE, affording an *S*-to-*R* flip in the orientation of oxygen addition at C-15. Double oxygenation and cyclization to a prostaglandin endoperoxide are still possible in acetylated COX-2, albeit as a minor reaction and with oxygenation largely changed to the 15*R*-orientation. COX, cyclooxygenase; HETE, hydroxyeicosatetraenoic acid; PGH, prostaglandin H.

Aspirin affects COX catalysis by transfer of its acetyl group to the hydroxyl of Ser530 in the COX active site (54). Ser530 lines the fatty acid binding channel across from Tyr385, the residue that sparks substrate oxygenation by abstraction of a hydrogen atom from the *bis*-allylic methylene between the 11,12- and 14,15-double bonds of the AA substrate (55, 56). In the COX-1 isoform, acetylation of Ser530 blocks access of the fatty acid substrate to the active site/Tyr385, and the enzyme is inactive (57). In the COX-2 isoform, acetylation of Ser530 still allows fatty acid oxygenation to occur. The product of acetylated COX-2 with AA is 15*R*-HETE, with the configuration of the 15-hydroxyl opposite to the configuration in PGH_2_ (Fig. 6) (58). The inversion in configuration in response to Ser530 acetylation indicates a major rearrangement of the oxygenation mechanism. Abstraction of the hydrogen at C-13 by acetylated COX-2 occurs with the same stereoselectivity as in formation of PGH_2_ (59), indicating that binding of AA from the carboxylate at the entrance of the channel through C13 is similar and that the disturbance occurs in the upper part of the fatty acid binding channel that houses C15 and the methyl end (11, 60). Exactly how oxygen addition—in the absence or presence of the acetyl group at Ser530—is controlled in COX catalysis has been the topic of many investigations; for example (10, 11, 60, 61, 62, 63). Selectivity can be achieved by enzyme control over the movement of oxygen or the fatty acid chain or a shielding effect or a combination of several factors (64). A satisfactory explanation has not been reached, in large part because of the lack of an X-ray structure that would show the alignment of AA substrate in the acetylated COX-2 active site. The inability to produce the pertinent structure, in turn, might indicate that there is substantial movement of the fatty acid chain around C-15 when Ser530 is acetylated.

Derivatives of AA with modification near the carboxylate reacted with acetylated COX-2 to form the predicted C-15 hydroxylated products. For anandamide and 2-arachidonoylglycerol (2-AG), the C-15 configuration of 15-HETE amide or glycerol ester was not established, whereas 3- and 5-HETE were shown to yield the expected 15*R*-configuration diols (35, 40, 65, 66).

Acetylation of COX-2 is the only currently known mechanism by which 15*R*-oxygenation can be achieved, besides a COX in the Caribbean coral *Plexaura homomalla* that forms 15*R*-PGH_2_ through a Val/Ile point mutation (18, 67, 68). The activity of acetylated COX-2 in converting DHA to 17*R*-HDHA or arachidonate to 15*R*-HETE has evolved to link with LOX transformations in producing dihydroxy and trihydroxy oxylipins with 17*R*/15*R*-hydroxy stereochemistry. Currently, over 500 references include the “aspirin-triggered” moniker as exemplified here in a few well-cited articles (69, 70, 71). The term “aspirin triggered” is used to specify the 15*R* stereochemistry in arachidonate and at the equivalent ω6 position in other potential substrates and, paradoxically, appears in product biosynthesis in the absence of any contact with aspirin (*e*.*g*., (72)).

The understanding of 15*R*-HETE as the sole product of aspirin-acetylated COX-2 was revised after a careful analysis of polar products that were formed by the acetylated enzyme (73). The polar products contained a mixture of PGE_2_ and 15*R*-PGE_2_, with the latter in enantiomeric excess of 40% or 24%, depending on the method of analysis, thus suggesting enzymatic origin (Fig. 6). Though only about 15% in abundance compared with 15*R*-HETE, the formation of PGE_2_ and 15*R*-PGE_2_ by the aspirin-acetylated enzyme indicated that AA can align in the modified active site in a manner that allows PG formation to occur. Hydrogen abstraction, the initial oxygenation, and the two cyclization reactions proceed as in the unacetylated enzyme and up to the second oxygenation at C-15, which is affected as in formation of 15*R*-HETE. The second oxygenation at C-15 is less controlled with the prostanoid intermediate than when 15*R*-HETE is formed, indicating that the same position can be oxygenated with more or less stereocontrol. This might be due to the fact that in the prostanoid intermediate, with the carbon chain “shortened” through cyclization, C-15 is pulled toward the channel entrance and away from Tyr385 and thus sits at a different location in the channel than when 15*R*-HETE is formed, and this change in position likely drives a difference in how oxygen is attached (73).

The formation of 15*R*-configurated PGH_2_ contributes to a better understanding of catalytic and stereocontrol by acetylated COX-2. Whether 15*R-*PGs can be formed *in vivo* upon aspirin treatment has not been convincingly established, although 15*R*-PGD_2_ was shown to inhibit platelet aggregation, thus providing a potentially additional mechanism for the cardiovascular benefits of aspirin (73).

## Concluding remarks

Mechanistic studies using unusual or atypical fatty acid substrates or derivatives have expanded the spectrum of COX products beyond PGH_2_ to include PG esters and amides, 15*R*-HETE, HKs, and 5-OH-PGs, among others. Notably, these products were established first in biochemical *in vitro* transformations and then, reaching beyond enzymological insights, shaped the understanding of physiological roles of COX enzymes and their inhibition. Formation of atypical oxylipins in a (patho-)physiological context has spurred research into their biological effects, uncovering a role for COX-2 in maintaining levels of endocannabinoids (2-AG and anandamide) or in supporting angiogenesis (HKs) (44, 50).

Despite decades of mechanistic studies testing substrates and defining products of COX catalysis, key observations still lack adequate mechanistic explanations, including but not limited to the atypical reactions. Our understanding of how stereocontrol of oxygenation in PG biosynthesis is achieved is still largely descriptive. We know what substrates give what products and how mutations change catalytic outcome, but underlying chemical or biophysical effects are still elusive: for example, in 15*R*-HETE formation by acetylated COX-2, given that oxygen enters through the same channel as the fatty acid substrate (61): How does oxygen pass by C11, presumably a reactive site in the pentadienyl radical following H-abstraction at C13, to reach C15?—Does the enzyme shield the 11-position or prevent the radical from delocalizing there? Does acetylation of Ser-530 block 15*S* oxygenation or help expose the 15*R*-position? Does the enzyme twist the substrate/pentadienyl radical in a way that directs the reactivity and location of the radical?

The functional plasticity of the COX reaction, by reacting with unconventional substrates and producing equally unconventional products, has established a host of eicosanoids and oxylipins formed in parallel or in competition to AA-derived PGs. These studies have also helped to provide a much more granular picture of the unique effects of the drugs used to inhibit the COX enzymes, for example, with aspirin not only inhibiting thromboxane and other PGs but also triggering the formation of 15*R*-HETE and related oxylipins that may contribute to its pharmacological effects. Some nonsteroidal anti-inflammatory drugs have a unique potency in inhibiting 2-AG oxygenation by COX-2 with much greater potency than AA oxygenation (74). This may extend to pharmacological regulation of endocannabinoid levels *in vivo* (50). And finally, the activities of fish oil EPA and DHA in cardioprotection may be linked to their unique reactivity with the COX enzymes.

## Conflict of interest

C. S. is an editorial board member of the *Journal of Biological Chemistry*. The authors declare that they have no conflicts of interest with the contents of this article.
